# Genotyping of *Campylobacter jejuni* and prediction tools of its antimicrobial resistance

**DOI:** 10.1007/s12223-023-01093-5

**Published:** 2023-10-10

**Authors:** Nicol Strakova, Hana Michova, Ekaterina Shagieva, Petra Ovesna, Renata Karpiskova, Katerina Demnerova

**Affiliations:** 1 Veterinary Research Institute, Hudcova 296/70, Brno, Czech Republic; 2 Laboratory of Food Microbiology, Department of Biochemistry and Microbiology, University of Chemistry and Technology, Prague, Czech Republic; 3 Institute of Biostatistics and Analyses, Masaryk University, Brno, Czech Republic; 4 Department of Public Health, Medical Faculty, Masaryk University, Brno, Czech Republic

**Keywords:** cgMLST, MLST, mP-BIT, PFGE, Antimicrobial resistance, ResFinder, RGI

## Abstract

Although *Campylobacter jejuni* is the pathogen responsible for the most common foodborne illness, tracing of the infection source remains challenging due to its highly variable genome. Therefore, one of the aim of the study was to compare three genotyping methods (MLST, PFGE, and mP-BIT) to determine the most effective genotyping tool*. C. jejuni* strains were divided into 4 clusters based on strain similarity in the cgMLST dendrogram. Subsequently, the dendrograms of the 3 tested methods were compared to determine the accuracy of each method compared to the reference cgMLST method. Moreover, a cost-benefit analysis has showed that MLST had the highest inverse discrimination index (97%) and required less workflow, time, fewer consumables, and low bacterial sample quantity. PFGE was shown to be obsolete both because of its low discriminatory power and the complexity of the procedure. Similarly, mP‑BIT showed low separation results, which was compensated by its high availability. Therefore, our data showed that MLST is the optimal tool for genotyping *C. jejuni*. Another aim was to compare the antimicrobial resistance to ciprofloxacin, erythromycin, and tetracycline in *C. jejuni* strains isolated from human, water, air, food, and animal samples by two gene sequence-based prediction methods and to compare them with the actual susceptibility of *C. jejuni* strains using the disc diffusion method. Both tools, ResFinder and RGI, synchronously predict the antimicrobial susceptibility of *C. jejuni* and either can be used.

**Supplementary Information:**

The online version contains supplementary material available at 10.1007/s12223-023-01093-5.

## Introduction

*Campylobacter jejuni* is continuously marked as the most frequently reported cause of foodborne gastrointestinal infections in humans in the EU, and it has been so since 2007. Recent statistics performed by the European Food Safety Agency (EFSA) for the year 2021 reported an average campylobacteriosis incidence of 41.1 cases per 100,000 inhabitants with the highest number of cases continuously reported by the Czech Republic (152.4 cases per 100,000 inhabitants) (ECDC and EFSA 2022). In 2021, the number of confirmed human cases of campylobacteriosis was 127, 840 in EU, a slight increase since 2020. The trend for campylobacteriosis in humans remained stable during 2017–2021, and over 10,000 campylobacteriosis cases were hospitalised in 2021 in the EU (ECDC and EFSA 2022). More than 80% of campylobacteriosis cases are caused by *C. jejuni*. Other species, such as *C. coli*, *C. fetus*, *C. upsaliensis*, and *C. lari*, have been less frequently reported in patients with gastroenteritis (ECDC and EFSA 2022). The most common sources for the strong evidence campylobacteriosis foodborne outbreaks are broiler meat and unpasteurised milk. Twenty outbreaks were reported with strong evidence and 229 with weak evidence in 2021 in the EU. Approximately 30% of acute enteritis cases develop a severely debilitating irritable bowel syndrome or the autoimmune disorders represented by Guillain-Barré syndrome and reactive arthritis, with an estimated mortality of approximately 5 deaths per 100,000 cases (Pope et al. 2007; Taha-Abdelaziz et al. 2023).

*C. jejuni* is the leading cause of bacterial gastroenteritis, and its monitoring can function as early warning signals for outbreaks and detect long-term changes in the bacterial population, such as emerging antimicrobial resistance (Nennig et al. 2021). In general, genotyping of microorganisms is very important in evaluating the global evolution of the pathogens and studying their genetic relatedness to determine their point source during epidemiological investigations (Blanc 2004; Wang et al. 2015). So far, several methods have been developed to monitor intra-species diversity of bacterial pathogens. To establish the suitability of a typing scheme, a number of factors should be evaluated, such as discriminatory power of the method, its reproducibility, possibility of standardization, and need of specialized equipment and skills of the technicians. The genotyping of campylobacter circulating in food, livestock, environment, surface, and wastewater is an important step in reducing the incidence of campylobacters in humans. Therefore, the comparison of genotyping methods was performed with aim to find the most reliable method. The selected 4 methods based on different principles were used for genotyping of *C. jejuni* strains, core genome multilocus sequence typing (cgMLST), multilocus sequence typing (MLST), pulsed field gel electrophoresis (PFGE), and multiplex PCR binary typing (mP-BIT). Currently, the highest and the most reliable discrimination of the isolates can be achieved by whole-genome sequencing (WGS). This high-resolution method is increasingly used for the epidemiological investigations of various pathogens, including *C. jejuni*. Therefore, cgMLST has been used as the reference method in our study. MLST is a simple sequencing method, PFGE uses digestion of DNA with restriction enzymes followed by separation and movement of DNA fragments in agarose gel, whereas the last method, mP-BIT, is a multiplex PCR based on detecting 18 variable elements distributed in the genome of *C. jejuni*.

In humans, campylobacter infections are usually self-limiting, although bacteraemia is more common among the elderly, immunocompromised people, and children (Thomas et al. 2020). On the other hand, the treatment of campylobacteriosis is based on antibiotic therapy. The first-line antimicrobial therapy for this disease includes fluoroquinolones (e.g. ciprofloxacin), macrolides (e.g. erythromycin), and tetracyclines (e.g. tetracycline) (EUCAST 2022). Antimicrobial resistance is a global health issue that involves the transfer of bacteria and genes between humans and animals (Reddy and Zishiri 2017). These complications together with the high frequency of the infections are the reasons why campylobacteriosis is ranked as one of the diseases with the highest economic burden (Batz et al. 2012; Gibney et al. 2014; Mangen et al. 2015). To address this concern, this study predicted the resistance of *C. jejuni* of different origins to ciprofloxacin, erythromycin, and tetracycline.

**Fig. 1:**
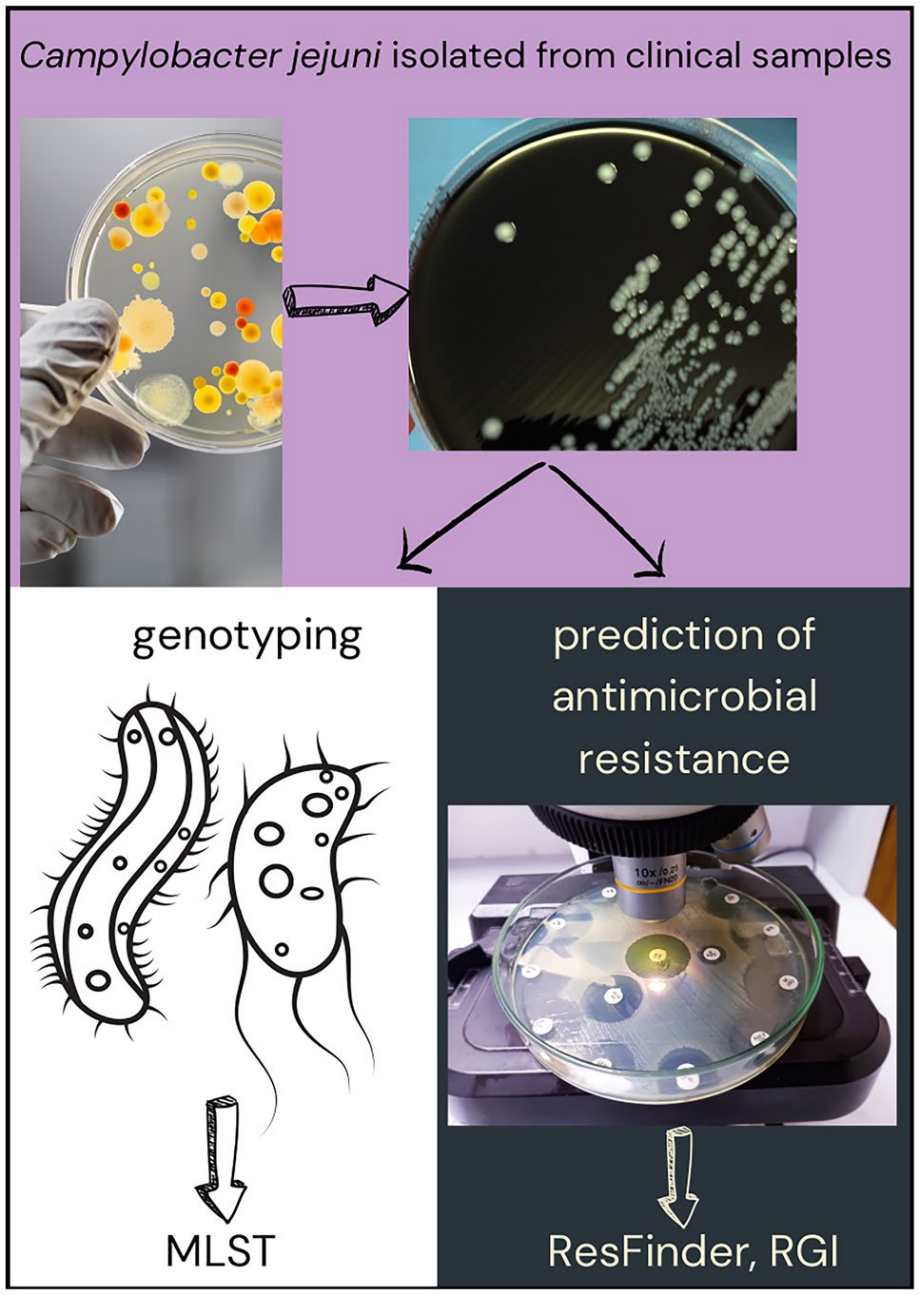
Genotyping of *Campylobacter jejuni* and prediction tools of its antimicrobial resistance. MLST can be used for campylobacter genotyping in routine practice. ResFinder and RGI, similarly predicted *C. jejuni* antimicrobial susceptibility and both prediction tools can be used to predict antimicrobial resistance

In summary, genotyping and antimicrobial resistance were evaluated in *C. jejuni* strains isolated from clinical, water, air, soil, animal and food samples. This study compared and selected the optimal genotyping method and method for prediction of antimicrobial sensitivity with the aim to find the optimal baseline methods for typing of *C. jejuni* strains in routine practice (see Fig. 1).

## Materials and methods

### Strain collection

The study was conducted on a cohort of *C. jejuni* strains isolated from three different niches, namely, humans, environment, and animals, between 2018 and 2019 (Table S1). Human strains (*n* = 11) were isolated from rectal swabs provided by clinical laboratories in anonymized form. The environmental *C. jejuni* strains (*n* = 14) were isolated from water, soil, and air samples. The most difficult was the collection of campylobacters from water samples. Campylobacters can be easily detected in water by non-cultivation methods in easy way, albeit without the possibility of further genotyping, storage or their sensitivity/resistance to antimicrobials (Van Dyke et al. 2010). Therefore, extensive water sampling from municipal wastewater treatment plants and surface waters, mainly ponds and lakes in different locations, was performed as was in detail described in our previous study (Strakova et al. 2021). Such strains of *C. jejuni* that were difficult to obtain from water (*n* = 12) were supplemented by strains isolated from soil, air, and animals. Two strains of *C. jejuni* were isolated from boot swabs and air filters. These strains were obtained during sampling on a poultry farm and complemented the group of environmental *C. jejuni* strains (Table S1). The animal strains (*n* = 10) were isolated from chicken cloacal swabs, raw chicken meat, and poultry offal. Animal swabs were obtained anonymously from slaughterhouses. The number of human and animal *C. jejuni* strains was approximately equal. The identity of *C. jejuni* strains isolated from human, environmental, and animal groups was verified by species-specific multiplex PCR and time-of-flight laser desorption/ionization mass spectrometry (MALDI-TOF/MS) as has been described previously (Kolinska et al. 2008; Strakova et al. 2021). The strains were stored at − 80 °C, and prior to every experiment, they were resuscitated by 72-h cultivation at 42 °C under a microaerobic atmosphere on mCCDA agar plates (Oxoid, UK).

### Extraction of bacterial DNA

Bacterial DNA was extracted either by a standard thermal lysis for PCR identification and mP-BIT or by blood and tissue kit (Qiagen, Germany) for WGS analysis. The concentration of DNA was measured using a Qubit fluorometer (Thermo Fisher Scientific, USA) with the Qubit dsDNA broad range kit (Thermo Fisher Scientific, USA). The purity of DNA was evaluated using NanoDrop (Thermo Fisher Scientific, USA). Only the samples with purity of A_260/280_ ≥ 1.8 and A_260/230_ ≥ 2.0 were taken into consideration. DNA was stored at − 20 °C in aliquots of 200 μL.

### Whole-genome sequencing (WGS)

WGS was performed in all *C. jejuni* isolates (*n* = 35). Preparation of DNA libraries by the Nextera XT DNA Library Preparation Kit (Illumina), and sequencing on the Illumina platform was carried out externally by LGC Genomics GmbH (Berlin, Germany). The quality of the reads was checked by LGC Genomics GmbH and summarized in the FastQC report. Phred score greater than 30 was set as short-reads quality criterion.

### Multilocus sequence typing (MLST)

The MLST analysis is based on the sequence comparison of the following seven housekeeping genes: *aspA* (aspartase A), *glnA* (glutamine synthetase), *gltA* (citrate synthase), *glyA* (serine hydroxymethyltransferase), *pgm* (phosphoglucomutase), *tkt* (transketolase), and *uncA* (ATP synthase a subunit) (Dingle et al. 2001). WGS data were used for MLST. The sequences of the housekeeping loci were detected using Ridom SeqSphere+ software. The sequence types (STs) and clonal complexes (CC) were assigned according to the MLST website http://pubmlst.org/campylobacter.

### Pulsed-field gel electrophoresis with macrorestriction analysis (PFGE)

The PFGE analysis was performed according to the standardized laboratory protocol recommended by PulseNetUSA using the SmaI endonuclease (Tolar et al. 2019). Profiles were analysed using BioNumerics 6.6 fingerprinting software (Applied Maths, Belgium) with clustering based on Dice Similarity Index. The relationships of the isolates were evaluated from the resulting dendrogram, and pulsotypes were compared with the PulseNet database to identify potential commonly occurring types.

### Multiplex PCR binary typing (mP-BIT)

mP-BIT typing was performed using the KAPA multiplex PCR kit (Kapa Biosystems, USA) with master mix and amplification set according to the manufacturer’s instructions (Yamada et al. 2015). In detail, two multiplex PCRs were performed with 18 designed primer sets (Table S2). Detection of *hipO* gene was used for *C. jejuni* strain verification. PCR products were separated by 3% agarose gel electrophoresis (Bio-Rad Laboratories, USA) in Tris borate EDTA at 100 V for 90 min, stained using Midori Green Advanced (Nippon Genetics Europe GmbH, Germany), and then visualized with UV transilluminator (Quantum Vilber Lourmat, France). Band sizes were estimated by comparison with a 50 bp molecular size marker (New England Biolabs, USA). The results were then converted to a binary code, where the positive result was marked with “1” and the negative result with “0”. The binary codes were then converted into decimals representing the mP-BIT profiles.

### Core genome multilocus sequence typing (cgMLST)

cgMLST sequence typing based on comparison of 637 loci used WGS analysis (https://www.cgmlst.org/ncs/schema/145039/). The assembled sequence data were integrated in Ridom SeqSphere+ software and were further analysed as described below.

### Data processing

As mentioned above, most of the data were analysed by Ridom SeqSphere+ software (version 6.0.2; Ridom GmbH, Münster, Germany). WGS raw sequence data were assembled de novo using the Velvet assembler version 1.1.04 and are publicly available at NCBI database https://submit.ncbi.nlm.nih.gov (BioProject No. PRJNA 800 229). All WGS-based analyses were performed at default setting with reference sequence (genome of the strain NCTC 11168) identity of at least 90% and base sequence identity set to 99%. Genetic diversity of *C. jejuni* isolates was evaluated in forms of minimum spanning trees (MST) and phylogenetic trees (UPGMA). Data from cgMLST, MLST, PFGE, and mP-BIT analyses were evaluated separately for *C. jejuni* typing. The cgMLST, MLST, and mP-BIT dendrograms were prepared by Ridom SeqSphere+ software. PFGE dendrogram was prepared using BioNumerics 6.6 fingerprinting software (Applied Maths, Belgium). A cluster was defined as maximum differences of 1 allele for MLST, 13 alleles for cgMLST, and Dice Similarity Index ≥ 85% for PFGE and mP-BIT.

### Comparison of the methods

The dendrograms obtained by the particular typing schemes were visually compared, marking the grouped isolates with 4 colours (green, blue, yellow, and pink). The data of grouped strains were used to calculate discrimination index (Ebel and Frisbie 1991), and inverse discrimination index (Inverse DI) was counted for comparison of the tested methods. cgMLST was used as the reference method.

### Antimicrobial sensitivity testing

Antimicrobial susceptibility screening was performed using a disk diffusion test according to EUCAST for *Campylobacter* spp. (EUCAST 2022). Briefly, grown colonies of *C. jejuni* were suspended in PBS to reach a turbidity of 0.5 McFarland. The suspensions were then spread on Mueller-Hinton agar supplemented with 5% of defibrinated horse blood and 20 mg/L β-NAD (BioRad, USA). After that, disks with desired antibiotics were placed on each plate (ciprofloxacin 5 μg, erythromycin 15 μg, and tetracycline 30 μg), which was then cultivated microaerobically 24 h at 42 °C The sensitivity/resistance of the strains was evaluated by the diameter of the inhibition zone. The antimicrobial sensitivity of aquatic strains has been described previously (Shagieva et al. 2021).

### Prediction of antimicrobial resistance

WGS data were used for prediction of antimicrobial resistance to ciprofloxacin, erythromycin and tetracycline. To do so, the data were analysed using two online tools based on different algorithms. First of them was ResFinder available at the website of the Center for Genomic Epidemiology https://cge.food.dtu.dk/sevices/ResFinder (Camacho et al. 2009; Zankari et al. 2017; Bortolaia et al. 2020). The second tool was Resistance Gene Identifier (RGI; https://card.mcmaster.ca/analyze/rgi) provided by the Comprehensive Antibiotic Resistance Database (CARD). The genes were evaluated by their homology and SNP and only those whose identity of matching region was higher than 95% were considered as present in the genome (Alcock et al. 2019).

### Ethical approval statement

Ethical approval was not required because the samples were anonymized by-products of routine clinical care.

## Results

We analysed a set of campylobacter strains isolated from human (*n* = 11; 31%), environmental (*n* = 14; 40%), and animal (*n* = 10; 29%) samples, and genotyping of the strains and their antimicrobial resistance was evaluated.

## Genotyping of *C. jejuni*

*C. jejuni* strains were independently genotyped by four methods, MLST, PFGE mP-BIT, and cgMLST, with the aim to find a reliable genotyping method available for the majority of laboratories. The results of these genotyping methods are summarized in detail in Table S1. This complex table contains the list of *C. jejuni* strains with their origin, source, date of isolation, sequence types (ST), pulsotypes, and mP-BIT profiles and in addition identification of the FASTA file in the public database and clonal complexes (CC). The genotyping was performed, and the results were displayed using the minimum spanning tree for the MLST method, using the PFGE dendrogram, using the binary code table obtained by the mP-BIT method, and finally using the minimum spanning tree generated by cgMLST, after which closely related strains were arranged into clusters.

### MLST

**Fig. 2:**
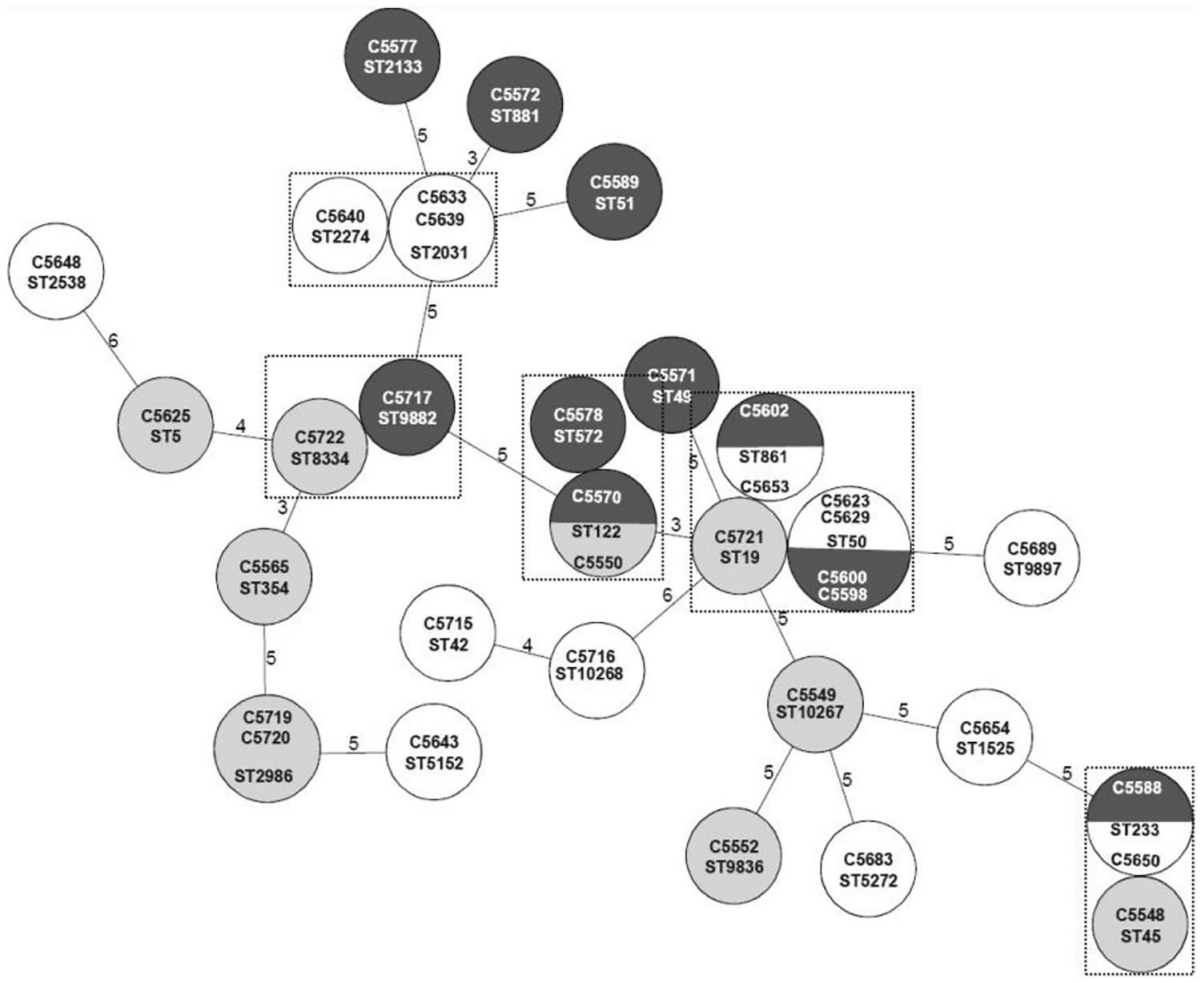
Minimum spanning tree of *C. jejuni* based on MLST analysis. The allelic distance, recorded on the lines, was evaluated according to the sequence types (STs; listed in the circles) by Ridom SeqSphere+. Strains are colour-coded by their origin (dark grey, human; light grey, animal; white, environmental). Clusters of strains differing in maximum one allele are marked by a dashed line

*C. jejuni* strains were characterized by conventional MLST using sequences of 7 housekeeping genes. Our results showed that 27 different STs were identified among the 35 examined strains, with ST50 being the most prevalent. Moreover, 20 strains were assigned into 6 clusters based on ST similarities, and 15 strains were marked as singletons (Fig. 2).

Furthermore, the occurrence of the same *C. jejuni* genotypes was compared with their origin. The biggest cluster contained 7 strains of different origins (3 human, 3 environmental, and 1 animal), while other clusters consisted of two to three strains. There was no clear pattern between the clustering and origin of the strains. The most frequent ST was ST50 (*n* = 4, 11%); two of these strains originated from a municipal wastewater treatment plant sampled in February and March (C5623 and C5629), and the other two were human clinical isolates (C5600 and C5598). Five STs were presented in duplicates in our dataset—ST122 (human C5570 and animal C5550), ST233 (human C5588 and environmental C5650), ST861 (human C5602 and environmental C5653), ST2031 (environmental C5633 and C5639), and ST2986 (animal C5719 and C5720).

### PFGE

**Fig. 3:**
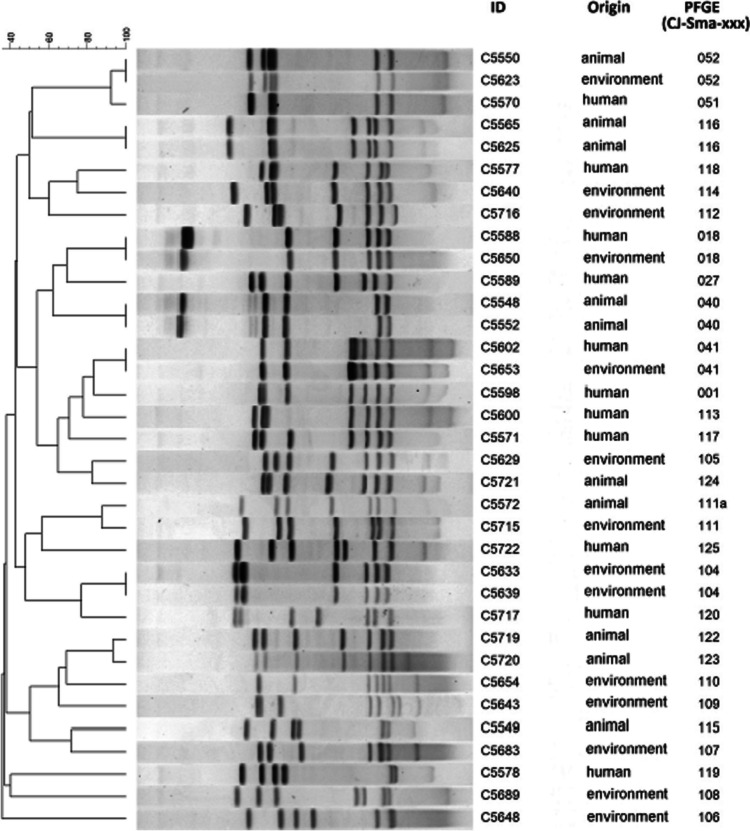
PFGE analysis of *C. jejuni* strains. The dendrogram with ID, origin, and pulsotypes of the strains

The PFGE analysis of *C. jejuni* strains showed 7 pulsotypes that contained two strains each: CJ-Sma-018 (human C5588 and environmental C5650), CJ-Sma-040 (animal C5548 and C5552), CJ-Sma-041 (human C5602 and environmental C5653), CJ-Sma-052 (environmental C5623 and animal C5550), CJ-Sma-104 (environmental C5633 and 5639), CJ-Sma-111 (human C5572 and environmental C5715), and CJ-Sma-116 (animal C5625 and C5565). In summary, PFGE classified our strains into 28 different pulsotypes, and 21 pulsotypes were unique in the sample collection (Fig. 3).

### mP-BIT

The mP-BIT analysis classified the *C. jejuni* strains into 25 different mP-BIT profiles. The most common mP-BIT profiles (File S1) were 15/311 (C5623, C5643, and C5570 strains) and 62/63 (C5629, C5600, and C5721 strains). These strains were isolated from environmental, human, and animal samples (Table S1). The mP-BIT profiles 10/49, 398/169, and 446/63 each occurred in two strains of animal origin. There are 20 mP-BIT profiles occurring only once each time (Table S3).

### cgMLST typing

**Fig. 4:**
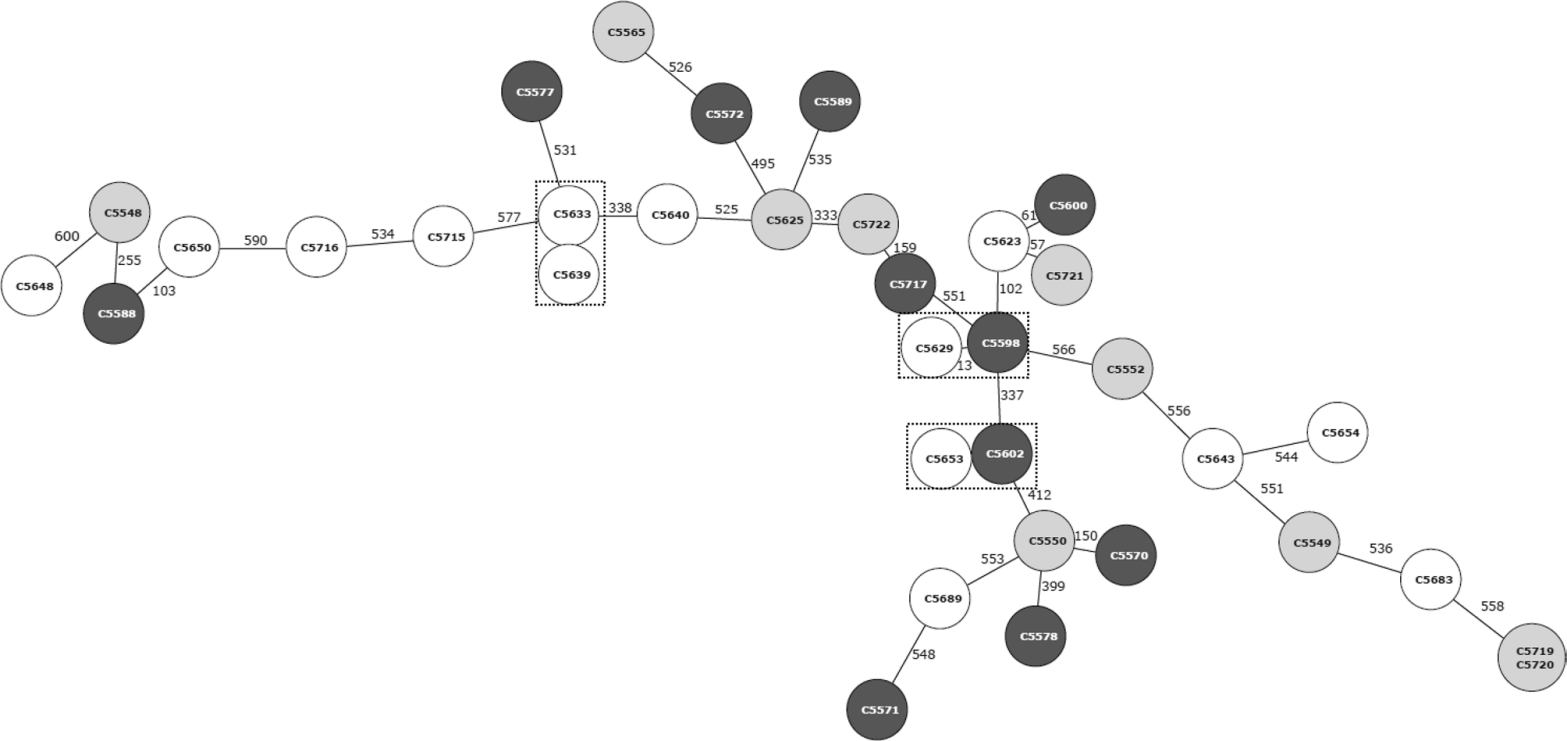
Minimum spanning tree of *C. jejuni s*trains based on cgMLST analysis by Ridom SeqSphere+ software. The circles are colour-coded based on the origin of the strains (human, dark; environmental, white; and animal, grey). Clusters of related strains differing in max. 13 alleles are marked by dashed lines

cgMLST analysis showed high diversity among the strains as they were distributed into 34 unique profiles (Fig. 4), while only one profile was shared by two strains (C5719 and C5720) isolated from two different samples of animal origin.

Next, clustering of cgMLST profiles showed the presence of three clusters (Fig. 4), each containing two strains (environmental origin C5633 with C5639, environmental origin C5629 with human origin C5598, and similarly environmental origin C5653 with human origin C5602) differing in maximum 13 alleles (99% of sequence identity). The remaining strains in our collection (*n* = 27) were singletons (Fig. 4).

### Comparison of the genotyping methods

**Fig. 5:**
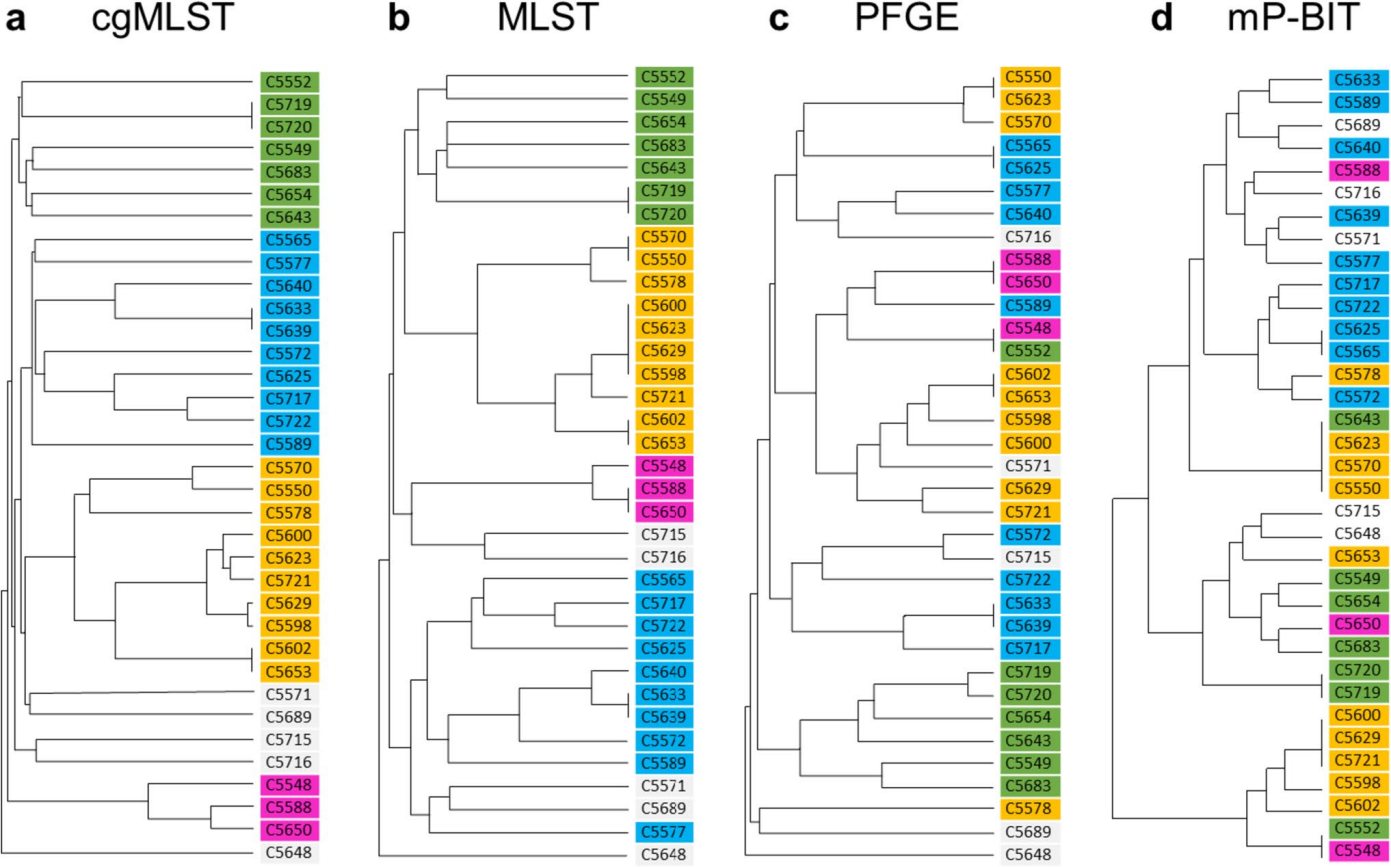
Comparison of *C. jejuni* strains. Relationship of strains was based on visual evaluation of dendrograms obtained from **a** cgMLST, **b** MLST, **c** PFGE, and **d** mP-BIT. Strains were grouped according to cgMLST dendrogram into four groups indicated by the same colour (green, blue, yellow, and pink), unrelated strains remained white

Two approaches were used to evaluate genotyping methods: (1) comparison of similarities in dendrograms and (2) cost-benefit comparison. The first approach compared the dendrograms of each methods with each other and evaluated inverse discrimination index (Inverse DI). This index was calculated in order to compare methods on the basis of similarity and relatedness of the bacterial strains. In the first approach, the strains were divided based on visual similarities into 4 clusters, green, blue, yellow, and pink (Fig. 5), using cgMLST as a reference method (Fig. 5).

Five of the 35 strains were outside any cluster and therefore remained of white colour. The green cluster was very compact and contained 7 *C. jejuni* strains, which were identically clustered by cgMLST (Fig. 5a) and MLST (Fig. 5b), while PFGE clustered 6 and mP-BIT 5 of the strains (Fig. 5c, d). The blue group (*n* = 10) was clustered by cgMLST and MLST, but it was divided in PFGE and mP-BIT dendrograms. The group contained two closely related strains of environmental origin (C5633 and C5639), which were marked as identical by cgMLST, MLST, and PFGE. The mP-BIT pulsotypes of these strains differed. The yellow group (*n* = 10) was again clustered by cgMLST and MLST dendrograms. However, the use of PFGE and mP-BIT fragmented the cluster into 3–4 smaller groups. The last and the smallest group (pink, *n* = 3) formed cluster only by cgMLST, MLST, and PFGE, but not by mP-BIT. Subsequently, the Inverse DI was calculated to statistically analyse the accuracy of the methods (Table 1). Our results showed that comparing to the reference method (cgMLST), one strain was classified differently when using MLST (Inverse DI 97%), and 10 strains were classified differently when using PFGE and mP-BIT (Inverse DI 71%).

**Table 1:** Comparison of the genotyping methods by Inverse Discriminatory Index (Inverse DI)

	No. of congruently assigned strains	Inverse DI
cgMLST	Reference (30*)	-
MLST	29/30	97%
PFGE	20/30	71%
mP-BIT	20/30	71%

cgMLST was used as a reference method. Five of the 35 strains were outside of any cluster and were excluded from the analysis

For the second approach, the genotyping methods were compared on the basis of the equipment needed for the analysis, the complexity of the workflow, the time needed for analysis, the laboratory costs of consumables, and the sample quantity and quality requirements (Table 2). The most expensive and time-consuming method, especially for conventional laboratories without their own sequencer, is cgMLST, which also requires strong bioinformatic skills for the evaluation of WGS data. In contrast, the simplest and the least expensive is mP-BIT, which can be easily performed in the majority of microbiology laboratories. MLST and PFGE are in the middle of the cost-benefit ratio.

**Table 2:** Complexity of requirements of the genotyping methods for the typing of cultivatable strains

	Equipment	Workflow	Time	Consumables	Sample
cgMLST	+ + + +	+ + + +	+ + + +	+ +	+ + +
MLST	+ + +	+ +	+ +	+	+
PFGE	+ +	+ + +	+ + +	+ + + +	+ + + +
mP-BIT	+	+	+	+ + +	+ +

cgMLST, MLST, PFGE, and mP-BIT were compared based on 5 parameters, e.g. complexity of the equipment requirement, the difficulty of the workflow, the time needed per test, the cost of laboratory solutions or kits, and the sample quantity. The range of lowest (+) and highest (+ +  + +) requirements for the tested methods are given for each parameter separately

Altogether, our results proved that MLST was the most reliable genotyping method. It had high Inverse DI (97%) and good cost-benefit ratio, too.

## Evaluation of antimicrobial resistance

The disk diffusion method was used to detect antimicrobial susceptibility of *C. jejuni* strains isolated from 3 primary niches (human, environmental and animal samples). Out of 35 isolates, 21 isolates were resistant to ciprofloxacin, 2 isolates were resistant to erythromycin, and 11 isolates were resistant to tetracycline (Table 3). Next, the lower part of Table 3 describes the antimicrobial resistance to any used antimicrobial, multidrug resistance (resistance to two or three antimicrobials) and the percentage of the total strain resistance. Moreover, WGS data were processed with two available tools (ResFinder and RGI) to predict their potential antimicrobial resistance of the strains. Our results showed that the strains displayed a high degree of antimicrobial resistance. Each of the prediction methods provided both false-positive and false-negative results. A detailed analysis of these software tools was performed.

**Table 3:**
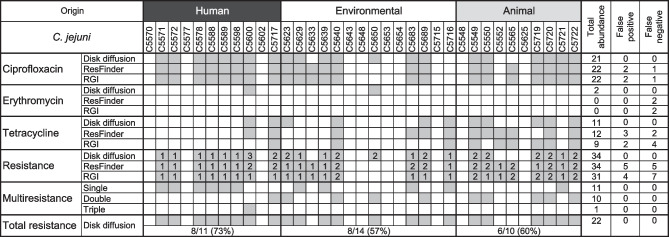
Antimicrobial resistance of *C. jejuni* strains

Columns: origin, total abundance of resistance, and false positive and negative resistance. Lines: phenotypic and genotypic resistance to ciprofloxacin, erythromycin, and tetracycline. Multidrug resistance to 1, 2, or 3 antimicrobials and total number of resistant strains

*C. jejuni* had the highest resistance to ciprofloxacin (*n* = 21; 60%). ResFinder/RGI gave 2 false positive and one negative response in strains C5633, C5565, and C5650, respectively. The both prediction tools gave same response (Table 3).

ResFinder and RGI showed that all tested strains were sensitive to erythromycin. However, disk disfuse method showed resistance in C5600 and C5650.

The resistance to tetracyclin was detected in 11 strains but predicted in 12 and 9 strains strains by ResFinder and RGI, respectively (Table 3).

Although ResFinder better predicted overall Campylobacter resistance (10 of 34 detected by the disc diffusion method), both tools very closely predicted *C. jejuni* antimicrobial susceptibility, and both can be used to predict ATB resistance.

In total, the human *C. jejuni* strains were the most resistant strains (73%). Less resistant were animal (60%) and environmental (57%) strains. The evaluation of antimicrobial resistance showed that 22 *C. jejuni* strains were resistant to any tested antimicrobial. Moreover, 10 strains were resistant to two antibiotics, and even *C. jejuni* strain (C5600), isolated from a patient rectal swab sample, was multidrug resistant to ciprofloxacin, erythromycin and tetracycline (Table 3).

## The occurrence of antimicrobial resistance in well-defined clusters of *C. jejuni* strains

**Fig. 6:**
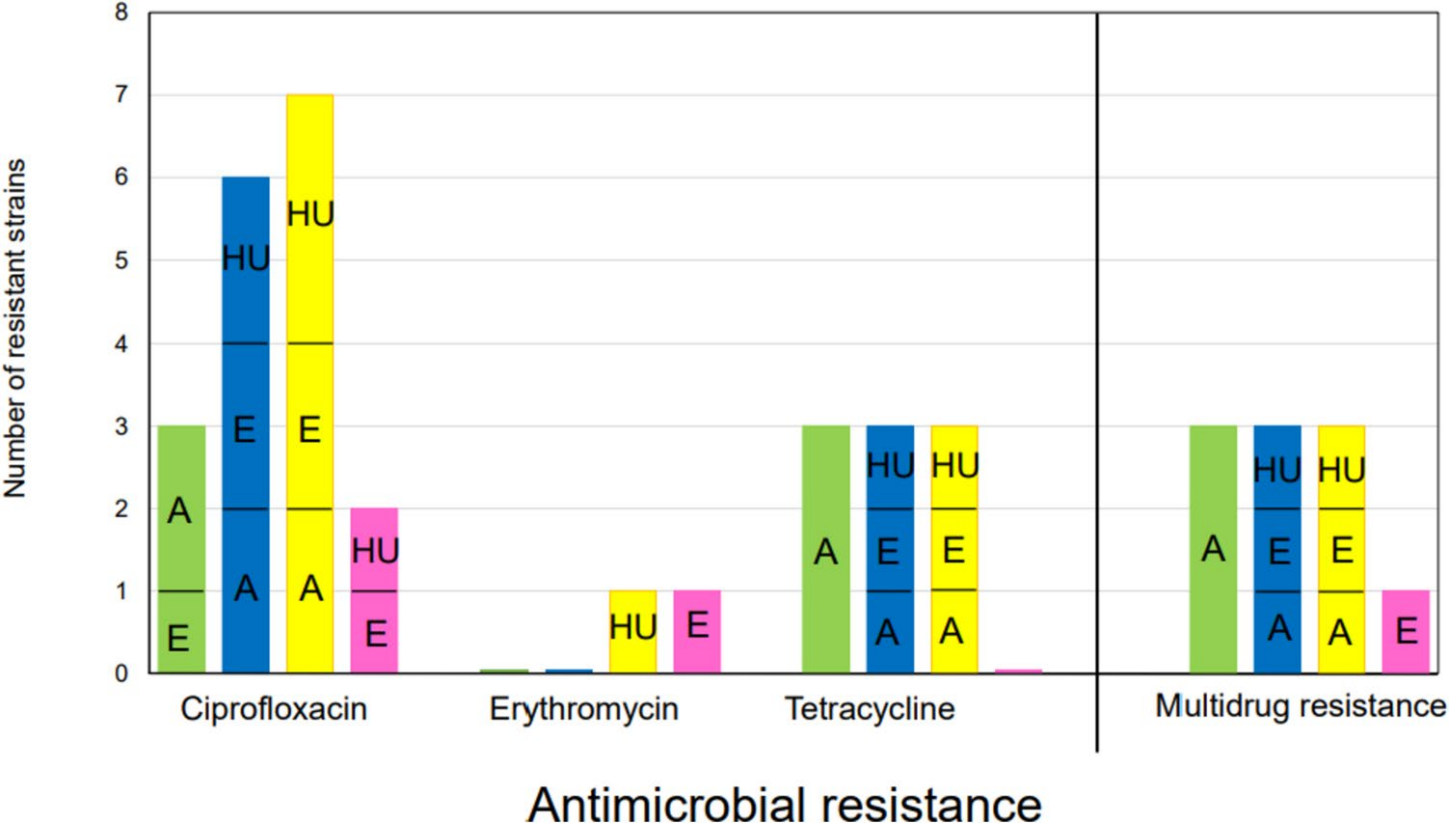
Antimicrobial resistance in well-defined clusters of *C. jejuni* strains. The occurrence of antimicrobial resistance to ciprofloxacin, erythromycin, and tetracycline, as well as multidrug resistance to at least 2 tested antimicrobials in each colour cluster

In the present paper, the occurrence of *C. jejuni*-resistant strains with resistance to ciprofloxacin, erythromycin, and tetracycline in coloured clusters was compared. Our results showed that resistance to ciprofloxacin occurred mainly in the yellow or blue clusters (15/35; 43%) (Fig. 5). No difference was found in the distribution of tetracycline-resistant strains in the green, blue, and yellow clusters. Moreover, resistance to at least 2 antimicrobials was also monitored. Our results showed that multidrug resistance was observed in 29% of *C. jejuni* (10/35), but there was no difference in the proportion among the green, blue, and yellow clusters (Fig. 5). In detail, the green cluster of multidrug-resistant strains contained only animal *C. jejuni* strains (C5549, C5719, and C5720) isolated from food samples such as goose liver and chicken meat (Fig. 5, Table 3). Multidrug-resistant strains of the blue cluster were isolated from a human rectal swab (C5717), an environmental sample isolated from wastewater (C5640), and animal *C. jejuni* isolated from chicken meat (C5722). Similarly, strains belonging to the yellow cluster were from a human rectum swab (C5600), wastewater (C5623), and goose liver (C5550). The lowest multidrug resistance was found in the pink cluster of strains (1/35; 3%) isolated from a surface water sample (Fig. 6, Table 3, Table S1).

## Discussion

As *Campylobacter jejuni* is continuously reported as the most common cause of zoonotic gastroenteritis, it is necessary to find reliable and accessible tools facilitating its epidemiological surveillance. With the rapid development of new techniques, it is important to critically evaluate both their advantages and disadvantages. In this work, we focused on the comparison of three commonly used genotyping methods based on different approaches, i.e. MLST, PFGE, and mP-BIT, to assess the phylogenetic proximity of *C. jejuni.* All methods were compared by their discrimination index, time consumption, overall costs, equipment requirements, complexity of the procedure, and quantity and quality of pure samples needed for the analysis. They were tested on a set of highly diverse strains of three different origins (human, environmental, and clinical), which were collected from multiple sources.

MLST and PFGE are currently the most common methods used for genotyping (EFSA Panel on EFSA Biological Hazards 2013). Both methods are time consuming and require specialized equipment and extensive experience and manual skills of the technicians. The results of PFGE can also be biased by rearrangement of the accessory genome (Romling et al. 1997). Routine surveillance could be performed with conventional MLST based on sequencing of 7 housekeeping genes. Its discriminatory power is high (only one of 30 strains was mismatched compared to cgMLST), while the price of the sequencing and simplicity of the data analysis make it affordable even for small laboratories. Our results showed that ST50 was the most prevalent sequence type in our study. It was in accordance with a current large study that compared 182 *C. jejuni* ST50 poultry isolates collected in 11 different countries (Wallace et al. 2021). They found that ST50 is the most frequently globally reported ST of *C. jejuni*, with numbers increasing each year. The second method was PFGE, which was until recently considered a gold typing standard, but the results are subjective and difficult to interpret (Leopold et al. 2014). It seems to be obsolete according to our study. The process is time consuming and requires specialized equipment, software, and skilled staff. In our work, the method correctly assigned only 20 out of the 30 strains compared to cgMLST.

The amplification method mP-BIT has been developed to provide useful epidemiological information for routine surveillance, outbreak investigations, and the molecular risk analysis of the isolates (Cornelius et al. 2014; Yamada et al. 2015). The typing is based on the analysis of 18 variable elements of the accessory genome, which play a role in survival, virulence, and antibiotic resistance of the strains and, therefore, could provide additional clinically relevant information. Since it is based on PCR, it can be performed simultaneously in a large number of strains, further reducing the time and cost of analysis. Our results showed that 20 out of 30 strains were correctly assigned by mP-BIT, similarly as by PFGE, although the authors of the method claimed that its discriminatory power exceeded that of PFGE (Yamada et al. 2015). However, due to the simplicity and cost of the process, it could still be used for basic genotyping in lower-income countries.

Currently, the highest and the most reliable discrimination of the isolates can be achieved by WGS (Hsu et al. 2020). Therefore, cgMLST was used as the fourth, reference genotyping method. This high-resolution method is increasingly used for the epidemiological investigations of various pathogens, including *C. jejuni* (Hsu et al. 2020). Several pitfalls of cgMLST need to be addressed. First, the default setting of the Ridom SeqSphere+ software (clustering based on similarity of 637 loci in maximum 13 alleles) was too strict for such a highly variable organism as *C. jejuni* is. cgMLST separated the 35 strains into 34 unique profiles. In our case, only 8 strains were clustered into four distinct groups, while the remaining 27 strains were marked as unrelated. Our results showed that strains of *C. jejuni* are structured into numerous distinct lineages due to high plasticity of the genome, extensive recombination, and high incidence of evolutionary diversity. However, after application of a more benevolent visual identification of a cluster, the strains were sorted into 4 larger groups that were then used as a base for the comparison of other methods by their dendrograms. This highlights one of the main disadvantages of cgMLST—the lack of international standardization of the bioinformatic analysis of the data, which is blocking the implementation of the method in the international molecular surveillance of *C. jejuni* (Nennig et al. 2021). Currently, three main typing schemes are broadly used for cgMLST of *C. jejuni*—the commercially available SeqSphere+ scheme used in this study, the Oxford scheme (Cody et al. 2017) based on the comparison of 1343 loci with a threshold being evaluated by the required discriminatory power, and the INNUENDO scheme (Rossi et al. 2018) using 678 loci with cut-offs set by a purpose of the typing to 4 (investigation of outbreaks), 59 (long-term monitoring), or 292 alleles (the highest compliance with the conventional MLST). Interestingly, even though the schemes are based on the comparison of core genome of *C. jejuni*, they only share 432 loci, probably due to vague definition of the “core genome” (Nennig et al. 2021). Another disadvantage against the use of cgMLST for broad genotyping is the availability and price of the analysis, as routine laboratories usually do not have direct access to WGS and there is a lack of staff skilled in bioinformatic analyses of the data. On the other hand, cgMLST also improves the linking of sporadic campylobacteriosis cases, which are more frequent than the outbreaks. Moreover, WGS data can provide additional information about their resistome, virulome, mobilome, and metabolome. Nevertheless, several factors must be taken into consideration prior routine use of cgMLST (Deneke et al. 2021), such as storage and interlaboratory exchange of large sequencing data, and a lack of standardized tools and workflows for their interpretation.

Several studies have described that *C. jejuni* populations in humans and chickens overlap (Sheppard et al. 2009; Wassenaar et al. 2009; Friis et al. 2010). Therefore, genotyping of campylobacters helps to monitor their occurrence in humans, animals, and the environment to reduce the number of campylobacter cases in humans. Moreover, *C. jejuni* can survive in poor environmental conditions (ECDC and EFSA 2022), and *C. jejuni* strains are generally more resistant to physiological stress than other bacteria strains (Sopwith et al. 2008). Recently, it has been found that *C. jejuni* can survive in aquatic environments (Nachamkin et al. 2008; Hyllestad et al. 2020; Gilpin et al. 2020; Shagieva et al. 2020; Strakova et al. 2022). Characterization of *C. jejuni* types in different reservoirs and environments can shed light on the epidemiology of campylobacteriosis in the area. As the world’s leading cause of human gastroenteritis, the food- and waterborne campylobacter needs to be intensively monitored through the One Health approach. Therefore, multiple strategies have been evaluated for reducing of campylobacter infections in humans, such as disinfection, decontamination, feed additives, and vaccination of animals during their lives or on carcasses (Taha-Abdelaziz et al. 2023). Although some of them have yielded promising results, none of these alternative approaches is as effective as antibiotics in treating campylobacter infections (Taha-Abdelaziz et al. 2023). However, acquired resistance to clinically important antimicrobials has compromised the effectiveness of antibiotic treatment over the past decades (Dai et al. 2020).

Fluoroquinolones, macrolides, and tetracyclines are antimicrobials recommended by EUCAST (2022) for campylobacter clinical breakpoint. Therefore, one representative antimicrobial for each recommended group was selected in the present study. Our results showed that ciprofloxacin belonging to fluoroquinolones exhibited the highest resistance (21/35; 60%). On the other hand, *C. jejuni* were the least resistant to the macrolide antimicrobial erythromycin (2/35; 6%). Macrolides are broad-spectrum antimicrobials that are commonly used in both humans and animals (Chibwe et al. 2023). Owing to the high incidence of resistance to fluoroquinolones among human isolates, macrolides have become the drugs of choice for human campylobacteriosis (Chibwe et al. 2023). Besides phenotype resistance, genotype resistance using two freely available online tools (ResFinder and RGI) was also used for prediction of antimicrobial resistance.

Surprisingly, 20 out of 21 ciprofloxacin and 6 out of 11 tetracycline-resistant strains were predicted correctly by all phenotype and genotype approaches. In general, all of the predictions contained both false-positive and false-negative results, leading to underestimation of the actual resistance. Correct prediction rates were similar for ResFinder and RGI: 85% for ciprofloxacin and 55% and 45% for tetracycline, respectively. ResFinder and RGI were not able to predict resistance to erythromycin. Our results are contrasting the recently published data describing correlation of prediction of antimicrobial resistance with the actual phenotype in *Salmonella enterica*, with detecting true positivity in 90–100% of the tested strains (Neuert et al. 2018; Bharat et al. 2022), but also in 516 clinical *C. jejuni* (Dahl et al. 2021). This may be due to the small number of strains in our study, and further confirmation experiments should be performed. Although WGS data can provide valuable information concerning the genome of the tested strains, the expected phenotype may be confirmed by cultivation methods.

## Conclusion

Currently, next-generation sequencing methods are in the main interest of genotyping methods. Despite their rapid development leading to greater availability and reduced costs, they are still inaccessible to routine laboratories, especially in lower-income countries. Our study showed that MLST can be successfully used for campylobacter genotyping because this available method provides the best discriminatory power and can be used for genotyping *C. jejuni* in routine practice. Moreover, ResFinder and RGI similarly predicted *C. jejuni* antimicrobial susceptibility, and both prediction tools can be used to predict antimicrobial resistance.

### Supplementary Information

Below is the link to the electronic supplementary material.

Supplementary file1 (DOCX 452 kb)

## Data Availability

Data is contained within the article.
