# Supplementary figures and images for: Genotyping of Campylobacter jejuni and prediction tools of its antimicrobial resistance
Source: Folia Microbiol (Praha). 2023 Oct 10;69(1):207–19. doi: 10.1007/s12223-023-01093-5 (PMC10876727; doi:10.1007/s12223-023-01093-5)

**Table S1, S2, and S3**


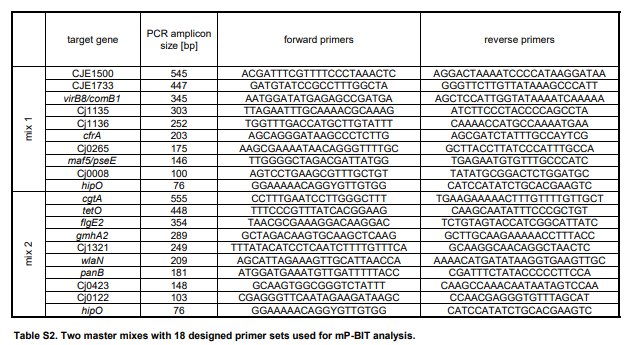

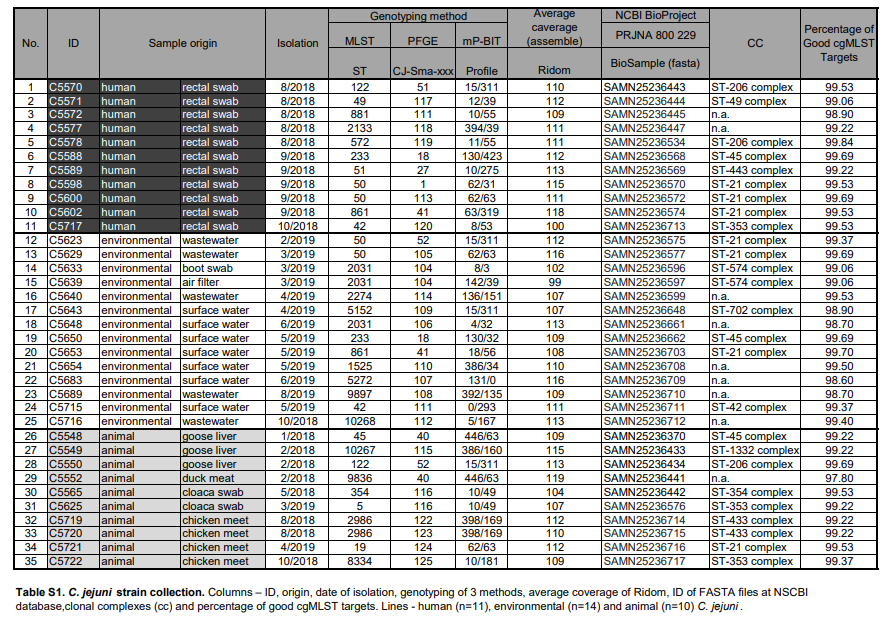


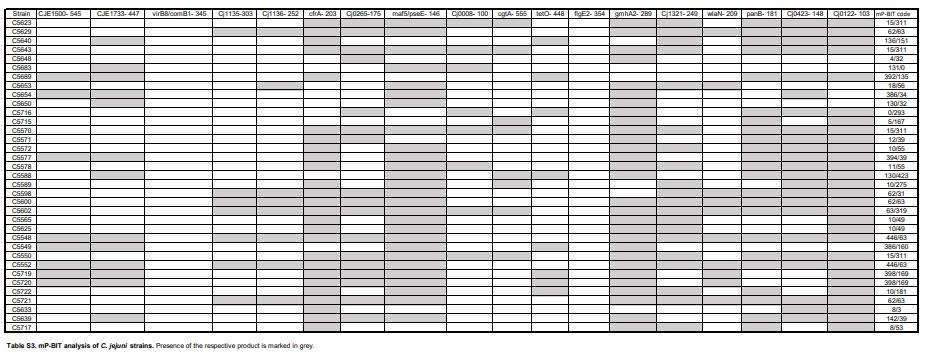

Supplement: Supplementary file 1 — Supplementary file1 (DOCX 452 kb) [file 12223_2023_1093_MOESM1_ESM.docx]
